# Low- or high-dose preventive aspirin use and risk of death from all-cause, cardiovascular disease, and cancer: A nationally representative cohort study

**DOI:** 10.3389/fphar.2023.1099810

**Published:** 2023-02-15

**Authors:** Yu Chen, Fuli Chen, Jie Liao, Hukui Han, Gang Li, Long Zhou

**Affiliations:** Department of Cardiology, Sichuan Provincial People’s Hospital, University of Electronic Science and Technology of China, Chengdu, China

**Keywords:** aspirin, cardiovascular diseases, cancer, mortality, cohort study

## Abstract

**Background and aim:** For a long time, aspirin has been recommended for the prevention of cardiovascular disease (CVD). However, results of long-term effects of aspirin use on the risk of CVD and all-cause death as well as cause-specific mortality are not consistent. This study aims to investigate the relationship between low- or high-dose preventive aspirin use and the risk of death from all-cause, CVD, and cancer among US adults aged 40 years and older.

**Methods:** A prospective cohort study was conducted by utilizing four cycles of the National Health and Nutrition Examination Survey (NHANES) and linked 2019 mortality files. Cox proportional hazard models accounting for multiple covariates were used to calculate hazard ratio (HR) and 95% confidence interval (CI) for the associations between low- or high-dose aspirin use and risk of death.

**Results:** A total of 10,854 individuals (5,364 men and 5,490 women) were enrolled in the study. During a median follow-up of 4.8 years, 924 death events including 294 CVD death and 223 cancer death were documented. We found no evidence that taking low-dose aspirin decreased the chance of dying from any cause (HR: 0.92, 95% CI: 0.79–1.06), CVD (HR: 1.03, 95% CI: 0.79–1.33), or cancer (HR: 0.80, 95% CI: 0.60–1.08). High-dose aspirin users had a higher risk of CVD death compared to participants who had never used aspirin (HR: 1.63, 95% CI: 1.11–2.41).

**Conclusion:** Using low-dose aspirin has no effect on the risk of death from any causes, whereas taking high doses of aspirin increases the risk of CVD death.

## Introduction

For a long time, people who have one or more risk factors for cardiovascular disease (CVD) have been recommended to take aspirin, one of the most often used drugs, to prevent CVD (Ittaman et al., 2014). According to estimates, 46.7% of US individuals aged 60 or older reported using aspirin to prevent CVD events (Liu et al., 2021). However, no consensus was established between the various guidelines committees on whether or how to use aspirin for primary prevention (Patrono and Baigent, 2019). According to the 2019 American College of Cardiology/American Heart Association Guidelines on the Primary Prevention of CVD, persons aged 40 to 70 who have a higher risk of developing CVD but not a higher risk of bleeding may benefit from using low-dose (75–100 mg/d) aspirin (Arnett et al., 2019). In contrast, the routine use of aspirin for the primary prevention of CVD in people without known CVD risk is not advised by the 2021 European Guidelines for Cardiovascular Disease Prevention in Clinical Practice (Visseren et al., 2022). The most recent recommendation statement from the US Preventive Services Task Force (USPSTF) indicates that persons aged 40 to 59 who have a 10% or greater 10-year CVD risk should consider taking low-dose aspirin as their primary method of preventing CVD, but that low-dose aspirin use is not recommended for adults 60 years of age or older (Davidson et al., 2022). The guidelines differ because there is limited or conflicting information about the benefits of using aspirin for primary prevention. There is a dearth of evidence in community-based general populations covering a wide age range, despite the fact that low-dose aspirin use was compared to placebo for its effectiveness in preventing CVD in particular populations. In addition to CVD events, the relationship between preventive aspirin use and mortality risk has also been widely concerned. However, no consensus was established between the various studies on this issue. For example, some studies found that low-dose aspirin use was associated with a lower risk of mortality (Chan et al., 2007; Skriver et al., 2019) and other studies have found that aspirin has no effect or even harmful effect on the risk of death (Del et al., 2022; Shahrivar et al., 2022). By utilizing the National Health and Nutrition Examination Survey (NHANES) and linked mortality data, this study aims to investigate the relationship between low- or high-dose preventive aspirin use and the risk of all-cause, CVD, and cancer deaths among US adults 40 years of age and older.

## Methods

### Study population

**FIGURE 1:**
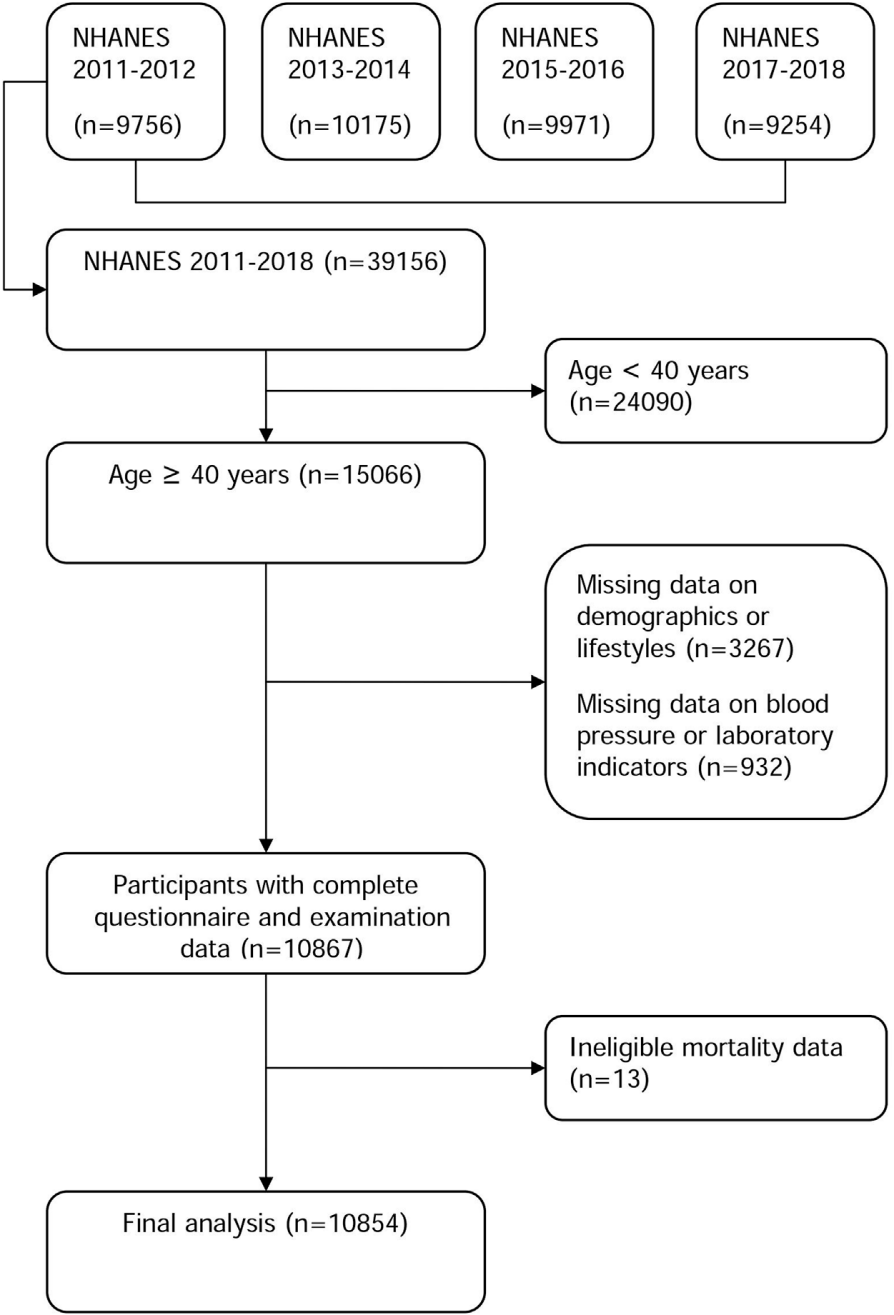
Flowchart of the study population selection.

Four NHANES cycles (2011-12, 2013-14, 2015-16, and 2017-18) were used to identify participants in this study. NHANES is a cohort of US residents who reside in communities and is nationally representative. A detailed description of the study population and sample design can be found elsewhere (Centers for Disease Control and Prevention, 2014). Within four cycles of the NHANES, 39,156 people nationwide were enrolled in the study (9,756 in the 2011-12 cycle, 10,175 in the 2013-14 cycle, 9,971 in the 2015-16 cycle, and 9,254 in the 2017-18 cycle). We only included individuals in this study who were 40 years of age or older (*n* = 15,066), as the questions about preventive aspirin use were relevant to this age group. In addition, we excluded 4,212 persons missing demographic or lifestyle information (*n* = 3,267), anthropometric results or laboratory markers (*n* = 932), or mortality information (*n* = 13). This resulted in the final analysis including 10,854 participants. Figure 1 shows a flowchart outlining the process of participant selection step-by-step. The study protocol was approved by the National Center for Health Statistics (NCHS) institutional review board (Protocol #2011-17 and Protocol #2018-01), and written informed consent was provided by all participants. The data used in this study is available to the public through https://www.cdc.gov/nchs/nhanes/. The data mining technology can be found in previous studies (Yang et al., 2020; Wu et al., 2021).

#### Preventive aspirin use

The preventive aspirin use questions were asked by trained interviewers in the participants’ homes. Participants were questioned about whether their doctors had ever advised them to take aspirin for preventive. The participant’s actual aspirin dosage was recorded in the preventative aspirin use questionnaire. The aspirin doses ranged from 25 mg to 500 mg, despite the fact that the majority of survey participants were taking 81 mg or 325 mg. We used ≤100 mg/d as low-dose aspirin use in accordance with prior studies and recommendations (McNeil et al., 2018a; Davidson et al., 2022). As a result, we defined high-dose aspirin use in the analysis as aspirin dosage >100 mg/d.

### Assessment of mortality

The NCHS has linked data collected from a number of its population surveys, such as NHANES, with records of deaths from the National Death Index (NDI). For this study, the 2019 NCHS Public-Use Linked Mortality Files were used to obtain mortality data, including mortality status, cause of death, and follow-up duration for all included persons from the date of survey participation until death or 31 December 2019. The participant’s principal cause of death is stated on their death certificate as one of nine cause-specific death categories based on the International Classification of Diseases, 10th Revision (ICD-10) code. (Centers for Disease Control and Prevention, 2019). In the current study, cancer mortality was recorded as ICD-10 code C00-C97, while cardiovascular mortality included deaths from heart disease (ICD-10 code I00-I09, I11, I13, and I20-I51) or cerebrovascular diseases (ICD-10 code I60-I69). Additionally, all-cause mortality was used to describe deaths for any reason (Zhou et al., 2022).

### Measurement of covariates

In-person interviews with participants were conducted by qualified interviewers to collect information on their lifestyles and demographics. The participants’ ethnicity was divided into four groups (i.e., Hispanic, White, Black, and others). There are three levels of education (i.e., <high school, high school, and ≥high school). The original marital status variable contains never married, married, living with a partner, separated, widowed, and divorced. We combined the categories of married and cohabiting into one to simplify the analysis. The categories of divorced, widowed, and separated people were also combined for the same purpose. The family monthly poverty level index, which measures the ratio of monthly income to poverty, was adopted to reflect family economics. According to NCHS-recommended analytical procedures, this indicator was divided into three categories: 1.30, 1.31–1.85, and >1.85. Drinkers were defined as participants who had ingested alcohol at least 12 times in the year prior. Sedentary time is defined as all time spent seated, excluding time spent sleeping, and is a reflection of sedentary behavior.

Body weight (in kilograms) and height (in centimeters) were measured by trained staff using standard devices. Body mass index (BMI) is derived as weight in kilograms divided by height in meters squared. BMI ≥30 kg/m^2^ was used by the World Health Organization (WHO) Expert Consultation to define obesity (WHO Expert Consultation, 2004). Systolic blood pressure (SBP) and diastolic blood pressure (DBP) were measured three times with a mercury sphygmomanometer. The analysis used the average of three blood pressure values. Hypertension was considered as SBP ≥140 mmHg and/or DBP ≥90 mmHg and/or using antihypertensive medications (Zhou et al., 2019).

Blood samples were obtained and kept frozen (−20°C) until they were sent to the University of Minnesota for analysis. On a Roche Modular P chemistry analyzer, the enzymatic approach was used to detect total cholesterol (TC). Hypercholesterolemia was defined as TC ≥6.2 mmol/L and/or current drug use. High-performance liquid chromatography (HPLC) Glycohemoglobin Analyzer was used to measure hemoglobin A1c. Hemoglobin A1c ≥6.5% and/or ongoing insulin or hypoglycemic drug use were used to diagnose diabetes.

### Statistical analysis

For continuous variables that do not conform to the normal distribution, descriptive data on participant characteristics were shown as weighted median (p25-p75), and for categorical variables, frequency (weighted percentages) was used. Participants were divided into three groups based on how they had previously taken aspirin for preventive (i.e., no use, low-dose, high-dose). To compare the between-group differences in characteristics, we used Kruskal-Wallis method for continuous variables and a $\chi^{2}$ test for categorical variables. The hazard ratio (HR) and 95% confidence interval (CI) for the risks of dying from any cause, CVD, or cancer were calculated using Cox proportional hazards models. Age, sex, ethnicity, education levels, family poverty rates, marital status, smoking, drinking, time spent sitting down, obesity, hypertension, diabetes, and hypercholesterolemia were adjusted in the model. Additionally, we conducted a number of subgroup analyses based on age, sex, ethnicity, drinking, smoking, weight, diabetes, hypertension, and hypercholesterolemia. Considering that those with cardiovascular disease at baseline are more likely to take aspirin, we additionally eliminated participants who had ever received a diagnosis of heart failure, coronary heart disease, angina, heart attack, or stroke for the purposes of the sensitivity analysis. All analyses were performed using SAS 9.4 from the SAS Institute in Cary, North Carolina. The threshold for statistical significance was a two-tailed *p*-value of 0.05. The forest plot of the subgroup analysis was created using the R package “forestplot” in R version 3.6.3 (R Foundation for Statistical Computing, Vienna, Austria).

## Results

In our study, 10,854 participants (5,364 men and 5,490 women) with a median (range) age of 60.0 (50.0, 69.0) years were enrolled in the analysis. The study population’s characteristics based on its prior use of preventive aspirin are shown in Table 1. Overall, participants who have used aspirin for prevention tended to be older, more likely to be men, to be of the white race, to drink, to spend more time sedentary, and to have cardiometabolic diseases like obesity, hypertension, diabetes, and hypercholesterolemia than participants who have never used aspirin.

**TABLE 1:** Characteristics of the study population (*n* = 10,854).

Characteristics	Aspirin use			*p* values
	No use (*n* = 7,404)	Low-dose (*n* = 2,970)	High-dose (*n* = 480)	
Age, years	54.0 (46.0–63.0)	64.0 (57.0–72.0)	63.0 (56.0–72.0)	<0.0001
Men, n(%)	3,495 (46.2)	1,561 (50.6)	308 (65.6)	<0.0001
Ethnicity, n(%)				<0.0001
Hispanic	1,826 (12.4)	618 (8.3)	52 (3.8)	
White	2,887 (70.2)	1,365 (77.1)	284 (82.3)	
Black	1,617 (9.7)	681 (9.1)	100 (7.5)	
Asian or others	1,074 (7.7)	306 (5.6)	44 (6.4)	
Education, n(%)				<0.0001
Less than high school	1,656 (13.6)	676 (12.6)	116 (14.4)	
High school	1,645 (21.9)	701 (23.4)	128 (29.6)	
More than high school	4,103 (64.5)	1,593 (64.0)	236 (56.0)	
Family monthly poverty levels				<0.0001
≤1.30	2,428 (21.0)	891 (17.8)	152 (18.9)	
1.31–1.85	1,079 (11.1)	469 (12.0)	78 (12.1)	
>1.85	3,897 (68.0)	1,610 (70.2)	250 (69.0)	
Marital status, n(%)				<0.0001
Married/cohabitation	4,619 (67.8)	1775 (67.2)	282 (68.7)	
Divorced/widowed	2,073 (24.2)	981 (26.9)	162 (24.5)	
Never married	712 (8.0)	214 (6.0)	36 (6.8)	
Smoking status, n(%)				<0.0001
Current	1,405 (18.4)	454 (14.0)	106 (17.5)	
Former	1,954 (27.7)	1,084 (36.6)	197 (43.4)	
Never	4,045 (53.9)	1,432 (49.4)	177 (39.1)	
Drinker, n(%)	4,761 (71.6)	1852 (69.8)	320 (73.6)	<0.0001
Sedentary time, hours/d	6.0 (4.0–9.0)	6.0 (4.0–9.0)	7.0 (4.0–9.0)	<0.0001
Obesity, n(%)	2,946 (39.3)	1,348 (45.4)	235 (56.3)	<0.0001
Hypertension, n(%)	3,206 (37.4)	2,117 (65.5)	339 (69.7)	<0.0001
Diabetes, n(%)	1,195 (11.5)	1,100 (28.6)	143 (31.1)	<0.0001
Hypercholesterolemia, n(%)	2,387 (31.8)	1,843 (61.7)	283 (61.5)	<0.0001

Values are presented as weighted median (p25-p75) or frequency (weighted percentage) when appropriate.

The effects of aspirin use at low or high doses on the risk of mortality from any cause, cardiovascular disease, and cancer are shown in Table 2. High-dose aspirin users had a higher risk of dying from a CVD (HR: 1.63, 95% CI: 1.11–2.41) than those who had never used aspirin, even after controlling for age, sex, ethnicity, education levels, family monthly poverty levels, marital status, smoking, drinking, and sedentary behavior. High-dose aspirin use was not significantly linked with all-cause mortality (HR: 1.18, 95% CI: 0.93–1.50) or cancer mortality (HR: 1.10, 95% CI: 0.67–1.80). Aside from that, there was no proof that taking low-dose aspirin reduced the risk of dying from any cause (HR: 0.92, 95% CI: 0.79–1.06), CVD (HR: 1.03, 95% CI: 0.79–1.33), or cancer (HR: 0.80, 95% CI: 0.60–1.08).

**TABLE 2:** Multivariable-adjusted hazard ratio (HR) and 95% confidence interval (CI) of aspirin use associated with deaths from all-cause, cardiovascular disease (CVD), and cancer.

	Aspirin use		
	No use	Low-dose	High-dose
Person-years of follow-up	36,244	14,115	2,342
All-cause death			
No. of deaths	494	348	82
Mortality, per 1,000 person-years	13.6	24.7	35.0
Age- and sex-adjusted HR	1 (Reference)	0.96 (0.84–1.11)	1.33 (1.05–1.69)
Multivariable-adjusted HR	1 (Reference)	0.92 (0.79–1.06)	1.18 (0.93–1.50)
CVD death			
No. Of deaths	141	119	34
Mortality, per 1,000 person-years	3.9	8.4	14.5
Age- and sex-adjusted HR	1 (Reference)	1.09 (0.85–1.39)	1.80 (1.23–2.63)
Multivariable-adjusted HR	1 (Reference)	1.03 (0.79–1.33)	1.63 (1.11–2.41)
Cancer death			
No. Of deaths	129	75	19
Mortality, per 1,000 person-years	3.6	5.3	8.1
Age- and sex-adjusted HR	1 (Reference)	0.81 (0.60–1.08)	1.20 (0.74–1.96)
Multivariable-adjusted HR	1 (Reference)	0.80 (0.60–1.08)	1.10 (0.67–1.80)

Adjusted for age, sex, ethnicity, education levels, family monthly poverty levels, marital status, smoking status, drinking, sedentary time, obesity, hypertension, diabetes, and hypercholesterolemia.

We conducted a subgroup analysis by age group because the USPSTF provided various recommendations to those 40–59 years old and 60 years or older. While high-dose aspirin usage was linked to a greater risk of CVD death among participants aged 60 years and older (HR: 1.65, 95% CI: 1.10–2.47), low-dose aspirin use had no significant effects on all-cause and cause-specific mortality among any age group (Table 3).

**TABLE 3:** Stratified analysis for the association between aspirin use and risk of death by age.

	Aspirin use		
	No use	Low-dose	High-dose
Age between 40 to 59 years			
Person-years of follow-up	22,443	4,075	794
All-cause death			
No. of deaths	106	35	8
Mortality, per 1,000 person-years	4.7	8.6	10.1
Age- and sex-adjusted HR	1 (Reference)	1.41 (0.95–2.08)	1.63 (0.79–3.36)
Multivariable-adjusted HR	1 (Reference)	1.16 (0.77–1.75)	1.15 (0.55–2.39)
CVD death			
No. of deaths	20	12	2
Mortality, per 1,000 person-years	0.9	2.9	2.5
Age- and sex-adjusted HR	1 (Reference)	2.77 (1.31–5.85)	2.31 (0.53–10.04)
Multivariable-adjusted HR	1 (Reference)	1.61 (0.73–3.53)	1.31 (0.29–5.86)
Cancer death			
No. of deaths	28	6	4
Mortality, per 1,000 person-years	1.2	1.5	5.0
Age- and sex-adjusted HR	1 (Reference)	0.76 (0.31–1.86)	2.66 (0.92–7.68)
Multivariable-adjusted HR	1 (Reference)	0.86 (0.34–2.20)	2.51 (0.85–7.45)
60 years or older			
Person-years of follow-up	13,801	10,040	1,548
All-cause death			
No. of deaths	388	313	74
Mortality, per 1,000 person-years	28.1	31.2	47.8
Age- and sex-adjusted HR	1 (Reference)	0.93 (0.80–1.08)	1.30 (1.01–1.67)
Multivariable-adjusted HR	1 (Reference)	0.89 (0.77–1.04)	1.18 (0.92–1.53)
CVD death			
No. of deaths	121	107	32
Mortality, per 1,000 person-years	8.8	10.7	20.7
Age- and sex-adjusted HR	1 (Reference)	1.00 (0.77–1.30)	1.74 (1.17–2.57)
Multivariable-adjusted HR	1 (Reference)	0.97 (0.74–1.27)	1.65 (1.10–2.47)
Cancer death			
No. of deaths	101	69	15
Mortality, per 1,000 person-years	7.3	6.9	9.7
Age- and sex-adjusted HR	1 (Reference)	0.80 (0.59–1.09)	1.02 (0.59–1.76)
Multivariable-adjusted HR	1 (Reference)	0.80 (0.58–1.09)	0.93 (0.54–1.63)

Adjusted for age, sex, ethnicity, education levels, family monthly poverty levels, marital status, smoking status, drinking, sedentary time, obesity, hypertension, diabetes, and hypercholesterolemia.

**FIGURE 2:**
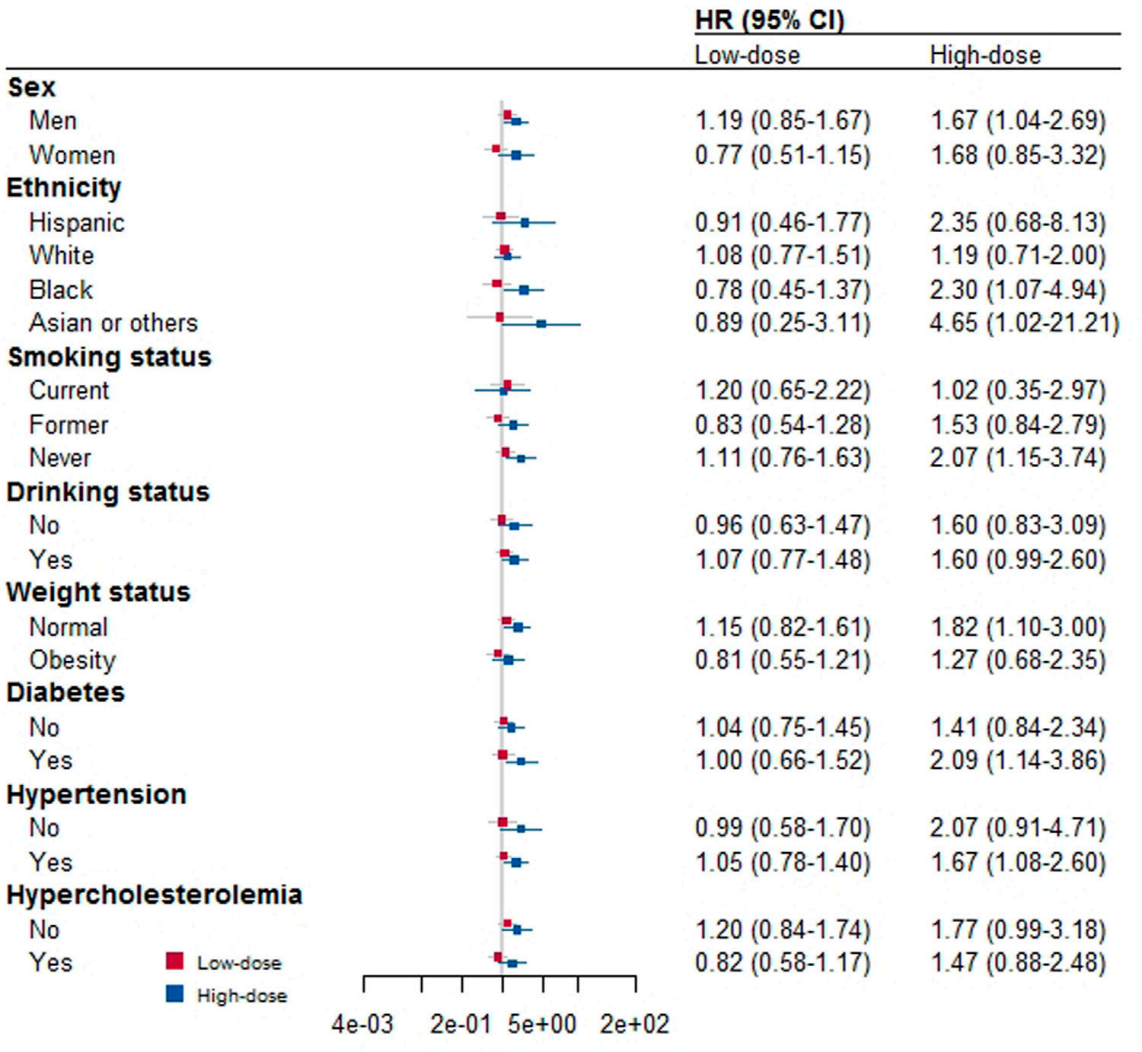
Stratified analysis for the association between aspirin use and risk of death by other covariates.

Figure 2 shows the results of additional stratified analyses we conducted by various factors to determine if low- or high-dose aspirin use is associated with CVD mortality. We found that low-dose aspirin use did not alter CVD mortality in any subgroups, which is consistent with our main finding. However, we found that using high doses of aspirin increased CVD mortality in male participants, those who were Black, Asian, or of other races, those who had never smoked, and those who were of normal weight, had diabetes, or had hypertension.

Results of sensitivity analysis by excluding those with CVD at baseline are shown in Table 4. Low- or high-dose aspirin use was not linked to cancer or all-cause death, which is consistent with our main findings. Low-dose aspirin use was not linked to CVD death, but high-dose aspirin use was positively associated with the hazard of dying from a CVD (HR: 2.04, 95% CI: 1.10–3.79).

**TABLE 4:** Sensitivity analysis for the association between aspirin use and risk of death by excluding participants who had cardiovascular diseases at baseline (*n* = 9,155).

	Aspirin use		
	No use (*n* = 6,769)	Low-dose (*n* = 2,106)	High-dose (*n* = 280)
All-cause death			
No. of deaths	349	189	35
Age- and sex-adjusted HR	1 (Reference)	0.95 (0.80–1.14)	1.38 (0.97–1.96)
Multivariable-adjusted HR	1 (Reference)	0.94 (0.78–1.14)	1.29 (0.90–1.83)
CVD death			
No. of deaths	79	57	12
Age- and sex-adjusted HR	1 (Reference)	1.20 (0.85–1.70)	2.00 (1.08–3.68)
Multivariable-adjusted HR	1 (Reference)	1.28 (0.89–1.83)	2.04 (1.10–3.79)
Cancer death			
No. of deaths	101	50	12
Age- and sex-adjusted HR	1 (Reference)	0.88 (0.62–1.24)	1.63 (0.89–2.99)
Multivariable-adjusted HR	1 (Reference)	0.89 (0.62–1.26)	1.53 (0.83–2.80)

Adjusted for age, sex, ethnicity, education levels, family monthly poverty levels, marital status, smoking status, drinking, sedentary time, obesity, hypertension, diabetes, and hypercholesterolemia.

## Discussion

In this 4.8-year median follow-up community-based prospective cohort study, we found that among participants aged 40 or older, high-dose aspirin use was linked to an increased risk of CVD death, whereas low-dose aspirin use was not linked to the risk of deaths from all causes, CVD, or cancer.

Aspirin’s role in the primary prevention of CVD has been the topic of heated discussion for many years. In 2018, the results of three fairly large randomized clinical trials (RCTs) with diverse populations and endpoints were published. In the ASPREE trial, 19,114 healthy older individuals (70 years of age or older) were enrolled. They were randomized to receive either a placebo (*n* = 9,589) or 100 mg of enteric-coated aspirin (*n* = 9,525). During a median follow-up of 4.7 years, the aspirin group had a higher risk of dying from any cause (HR: 1.14, 95% CI: 1.01–1.29) and dying from cancer (HR: 1.31, 95% CI: 1.10–1.56) than the placebo group. Aspirin use, however, had no discernible impact on CVD death when compared to placebo (HR: 0.82, 95% CI: 0.62–1.08) (McNeil et al., 2018b). In the ASCEND trial, 15,480 individuals with diabetes who were 40 years of age or older were randomized assigned to receive either a placebo or aspirin daily doses of 100 mg. After an average follow-up of 7.4 years, the aspirin group had a lower incidence of major vascular events than the placebo group (HR: 0.88, 95% CI: 0.79–0.97), including myocardial infarction, non-hemorrhagic stroke, and death from any vascular cause. However, the aspirin group experienced more serious bleeding episodes (HR: 1.29, 95% CI: 1.09–1.52) than the placebo group (Bowman et al., 2018). The ARRIVE trial enrolled 12,546 patients who were 55 or older in age and had a baseline moderate risk of CVD. A dose of 100 mg of aspirin (*n* = 6,270) or a placebo (*n* = 6,276) was given to participants, who were randomly selected. Time up to the first occurrence of cardiovascular mortality, myocardial infarction, unstable angina, stroke, or transient ischemic attack was the primary endpoint. After a median follow-up of 60 months, there was no discernible difference between the aspirin and placebo groups in the occurrence of the primary endpoint (HR: 0.96, 95% CI: 0.81–1.13) (Gaziano et al., 2018).

Aspirin use and the risk of death in individuals with cancer or hypertension have also been the topic of several observational studies. Per a *post hoc* analysis of the Systolic Blood Pressure Intervention Trial (SPRINT), aspirin use had no impact on the risk of all-cause death in hypertensive patients (HR: 0.84, 95% CI: 0.53–1.30) (Del et al., 2022). According to a cohort study using the Colorectal Cancer Data Base Sweden (CRCBaSe), aspirin use during follow-up was linked to an increased risk of all-cause mortality (HR: 1.09, 95% CI: 1.04–1.15) but not colorectal cancer (CRC) mortality (HR: 0.98, 95% CI: 0.91–1.06) among patients who were diagnosed with CRC (Shahrivar et al., 2022). In Denmark, after a prostate cancer diagnosis, the usage of low-dose aspirin was compared to death in a large cohort research. According to the findings, taking low-dose aspirin during exposure periods of 5 years (HR: 0.91, 95% CI: 0.83–1.00) and 7 years (HR: 0.84, 95% CI: 0.72–0.97) was associated with a slight reduction in prostate cancer mortality (Skriver et al., 2019). The Nurses’ Health Study found that women who reported low-to-moderate aspirin use had a decreased risk of death from all causes (HR: 0.75, 95% CI: 0.71–0.81), CVD (HR: 0.62, 95% CI: 0.55–0.71), and cancer (HR: 0.88, 95% CI: 0.81–0.96) during the course of a 24-year follow-up compared to those who never took aspirin (Chan et al., 2007).

As previously mentioned, aspirin use and the risks of cardiovascular events, overall mortality, and cause-specific death are still being debated. Previous RCTs and observational studies were mainly conducted in selected populations and there is a lack of studies conducted in community-based general populations. For the first time, our study provides a special perspective on aspirin’s primary preventive effect in the generally representative national population. We found that taking low-dose aspirin had no beneficial effect on the risk of dying due to any cause while high-dose aspirin use might increases the risk of CVD death, especially for those aged 60 years and older.

Bleeding is the most frequent aspirin side effect, which may cancel out any positive effects. For example, taking aspirin increased gastrointestinal bleeding incidents substantially (HR: 2.11, 95% CI: 1.36–3.28) compared with placebo in the ARRIVE trial (Gaziano et al., 2018). An earlier study found that aspirin users who are 70 years of age or older have a dramatically increased risk of bleeding events (Patrono et al., 2005). Aspirin’s positive and negative effects have the same underlying mechanism. The primary metabolite of arachidonic acid, thromboxane A2 (TXA2), can be inhibited by aspirin. Aspirin’s therapeutic effectiveness in reducing the risk of atherothrombosis and its side effect of bleeding can be explained by the fact that TXA2 is a potent inducer of platelet aggregation (Patrono, 2015; Petrucci et al., 2022). Controversial recommendations made by European and US guidelines reflect the uncertainty over the relative benefits and risks of using aspirin for the primary prevention of CVD.

Our study comes with a number of limitations. First, the usage of aspirin use was recorded only based a one-time questionnaire, which may lead to recall bias and inaccurate estimate of dose intensity. Second, although it is based on the fact that preventive drugs are generally taken regularly for a long time, the NHANES preventive aspirin use questionnaire did not collect participants’ drug duration, which should be taken into account when interpreting the results. Third, there is a lack of data on the incidence of CVD or bleeding events during follow-up because this analysis linked NHANES with death records of NDI Fourth, the observational study design makes it impossible to establish a causal link between aspirin use and risk of death.

In conclusion, using low-dose aspirin has no effect on the risk of death from any causes, whereas taking high dosage of aspirin use increases the risk of CVD death, especially for those aged 60 years and older.

## Data Availability

The raw data supporting the conclusion of this article will be made available by the authors, without undue reservation.
